# *Clostridium perfringens* Iota-Toxin: Structure and Function

**DOI:** 10.3390/toxins1020208

**Published:** 2009-12-23

**Authors:** Jun Sakurai, Masahiro Nagahama, Masataka Oda, Hideaki Tsuge, Keiko Kobayashi

**Affiliations:** 1 Department of Microbiology, Faculty of Pharmaceutical Sciences, Tokushima Bunri University, Yamashiro-cho, Tokushima 770-8514, Japan; 2 Institute for Health Sciences, Tokushima Bunri University, Yamashiro-cho, Tokushima 770-8514, Japan

**Keywords:** *Clostridium perfringens*, iota-toxin, ADP-ribosylating toxin, NAD^+^-glycohydrolase, ADP-ribosyltransferase, crystal structure, SN1 mechanism, internalization, endocytosis

## Abstract

*Clostridium perfringens* iota-toxin is composed of the enzyme component (Ia) and the binding component (Ib). Ib binds to receptor on targeted cells and translocates Ia into the cytosol of the cells. Ia ADP-ribosylates actin, resulting in cell rounding and death. Comparisons of the deduced amino acid sequence from the gene and three-dimensional structure of Ia with those of ADP-ribosylating toxins (ARTs) suggests that there is striking structural similarity among these toxins. Our objectives are to review the recent advances in the character, structure-function, and the mode of action of iota-toxin by consideration of the findings about ARTs.

## 1. Introduction

*Clostridium perfringens* is rod-shaped and gram positive. The microorganism is an anaerobic bacterium, but not a strict anaerobe. Strains of *C. perfringens* produce four major protein toxins, called alpha-, beta-, epsilon- and iota-toxins, which possess lethal and dermonecrotic activities at least, and are classified into five groups (type A to E) [1,2,3]. *C. perfringens*  type E, which produces alpha- and iota-toxins, causes antibiotic-associated enterotoxemia in rabbits, and is also implicated in sporadic outbreaks among calves as well as lambs [4]. *C. spiroforme* iota-like toxin is reported to be closely related to enteritis in rabbits [4]. Therefore, iota-toxin is highly likely to an important agent of enterotoxemia caused by *C. perfringens* type E.

Iota-toxin composed of an enzyme component (Ia) and a binding component (Ib) is a member of the binary toxin group [2,3,5,6] (Table 1 and Table 2). Significant progress has been made in the characterization of iota-toxin [5]. In addition, information on the biological properties, structure-function and mode of action of iota-toxin also has been accumulated [5]. This article summarizes current findings and deals with the mechanism of iota-toxin.

**Table 1:** Characterization of *Clostridium perfringens* iota-toxin.

Molecular weight	Iota a (Ia) 47,605 Da (413 residues)
	Iota b (Ib) 74,147 Da (664 residues)
Biological activity	Lethality, Dermonecrosis, Cytotoxicity
Enzymatic activity of Ia	NAD^+^-glycohydrolase (NADase)
	ADP-ribosyltransferase (ARTase)

**Table 2:** Structural organization of the binary toxin family.

Toxin	Enzymatic component	Binding component
C. perfringens Iota-toxin	Ia	Ib
B. anthracis Toxin	Lethal factor	Protective antigen
	Edema factor	
C. botulinum C2toxin	C2I	C2II
C. spiroforme Iota-like toxin	Sa	Sb

## 2. Characteristics of Iota-Toxin

*C. perfringens* iota-toxin was initially described by Bosworth in 1943 [7]. Later, the iota toxin was found to consist of two components [8], encoded by two genes in a plasmid, organized in an operon [9]. Two genes, with the same orientation, coding for Ia (454 amino acids) and Ib (875 amino acids) and separated by 243 noncoding nucleotides, were identified. It was reported that a predicted signal peptide (41 amino acids) and propeptide (13 amino acids) are missing from Ia and a predicted signal peptide (39 amino acids) and propeptide (211 amino acids) are missing from Ib [10], suggesting that the active Ia and Ib are composed of 400 and 664 amino acid residues, respectively. Alone, each component is nontoxic, but together, Ia and Ib are cytotoxic to various cultured cells, lethal to mice and dermonecrotic in guinea pigs [1,2,11] (Table 1).

**Figure 1:**
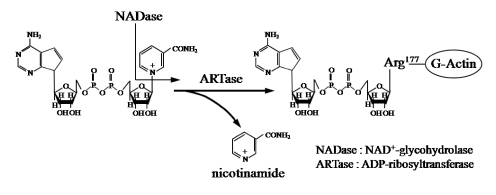
ADP-ribosylating activity of Ia.

**Figure 2:**
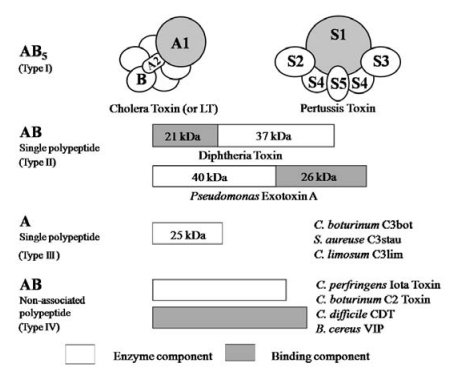
A family of bacterial ADP-ribosylating toxins.

Ia ADP-ribosylates skeletal muscle α-actin and nonmuscle β/γ-actin [2,3] (Figure 1). ADP-ribosylating toxins facilitate scission of the *N-*glycosyl bond between nicotinamide and the *N-*ribose of NAD (NAD^+^-glycohydrolase, NADase) and transfer the ADP-ribose moiety to target proteins (ADP-ribosyltransferase, ARTase) [5]. ADP-ribosylating toxins are classified into four families (Types I to IV) based on their respective targets [12,13] (Figure 2). Type I targets heteromeric GTP-binding proteins, and includes cholera toxin (CT) [14], pertussis toxin (PT) [15], and *Escherichia coli* heat-labile enterotoxin (LT) [16]. Type II [diphtheria toxin (DT) [17] and *Pseudomonas* exotoxin A (ETA) [18]] modifies elongation factor 2 (EF-2). Type III [*C. botulinum* C3 exoenzyme] ADP-ribosylates small GTP-binding proteins [19]. Type IV ADP-ribosylates actin [5]. Ia belongs to Type IV. Iota-toxin is a member of the binary toxin group, which includes *C. botulinum* C2 toxin (C2I and C2II) [20], *C. difficile* ADP-ribosyltransferase (CDTa and CDTb) [21], *C. spiroforme* toxin (Sa and Sb) [22] and *Bacillus cereus* vegetative insecticidal protein (VIP) [5]. The enzyme components of these toxins, which are structurally similar to Ia, are organized according to the classic A-B model.

**Figure 3:**
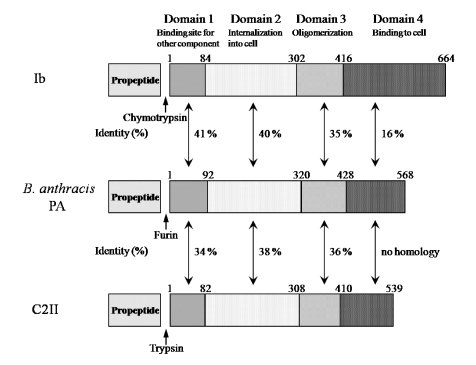
Comparison of amino acid sequence homology between Ib and PA or C2II.

Ib displays significant homology with the protective antigen (PA) of anthrax toxins (54.4% similarity overall) and C2II (39.0% similarity overall) [5] (Figure 3). The binding components of iota-toxin, anthrax toxin and C2 toxin similarly bind to receptors on membranes and interact with the enzyme components, mediating their entry into target cells, suggesting that they have a similar mode of action. As shown in Figure 3, the numbers reveal good homology in domains I, II and III forming the pore. In contrast, the homology in the receptor-binding domain IV is negligible among members of this group, which can be explained by the receptor-specificity of domain IV [5]. Therefore, the binding components of these binary toxins structurally seem to be conserved. 

Furthermore, Richard *et al.* reported that iota-toxin applied apically or basolaterally induces a rapid decrease in the transepithelial resistance (TER) of monolayers of CaCo-2 cells and disorganization of actin filaments as well as the tight and adherens junctions [23]. Treatment of Vero cells with iota toxin resulted in delayed caspase-dependent death. Unmodified actin did not reappear in toxin-treated cells, and Ia was detectable in the cytosol [24]. PA and C2II bind to receptors on cells and interact with edema factor or lethal factor and C2I, respectively, mediating entry into target cells [5]. Ib also binds to a receptor, interacts with Ia and transfers Ia into the cytosol. The activity of iota-toxin severely reduces the ability of actin to undergo polymerization, leading to disruption of the cytoskeletal architecture and cell death [5].

## 3. Structure and Function

Bacterial ADP-ribosylating toxins have been studied as agents that contribute to the pathogenesis of bacteria. Domenighini and Rappouli [25] classified ADP-ribosylating toxins into a CT group and a DT group, on the basis of amino acid sequences involved in the formation of the NAD-binding site and expected to play a role in the activity or in the folding of the proteins. In addition, the two groups do not share any detectable sequence homology.

**Figure 4:**
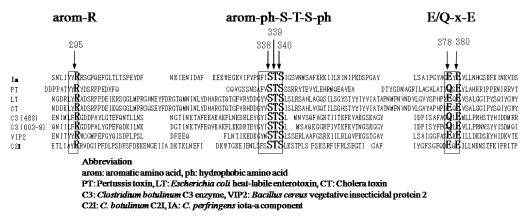
Conserved regions in ADP-ribosylating toxins.

Analysis of the LT crystal suggested that the nicotinamide ring of NAD^+^docks into the cavity [25]. ADP-ribosylating toxins of the CT group including LT, PT, *B. sphaericus* mosquitocidal toxin (MTX) [26], ExoS and C3 are known to contain three conserved regions, aromatic residue-R/H, E-X-E, and hydrophobic residue-S-T-S-hydrophobic residue, in the cavity formed by the β/α motif [25] (Figure 4). The role of these regions had been thought to be as follows. The polar side chains of E-X-E extend toward the catalytic cavity and are the common sequence involved in forming the NAD^+^ cleft, an aromatic residue-R/H located deep in the cavity binds NAD^+^, and the S-T-S consensus sequence is folded in a β strand representing the floor of the cavity [25]. Glu-378 and -380 in Ia are included within the E-X-E sequence essential for the enzymatic activity of CT, LT, and ExoS. Arg-295 is present in the aromatic residue-R/H sequence. Ser-338, Ser-340, and Thr-339 are present in the S-T-S consensus sequence [25]. A comparison of the amino acid sequence of Ia with sequences of these ADP-ribosylating toxins confirmed the existence of these motifs in Ia, showing that Ia belongs to the CT group.

Replacement of Arg-295 with alanine in Ia led to a complete loss of NADase, ARTase, lethal, and cytotoxic activities [27]. Perelle *et al.* [28] also reported that this residue is essential for ADP-ribosylating activity. Thus, it appears that the Arg-295 of Ia is equivalent to the Arg-7 of LT and Arg-299 of C2 toxin, which are required for ADP-ribosylating activity [29,30]. Replacement of Arg-295 with a basic amino acid such as histidine or lysine also caused a drastic reduction in NADase and/or ARTase activities, showing that Arg-295 can not be substituted with other basic residues. Therefore, it is apparent that the side chain of Arg-295 is essential for the NADase and ARTase activities of Ia [27]. Substitution of Arg-295 with lysine in Ia resulted in a significant reduction in the *Km* for NAD^+^, markedly reduced the *kcat* values for NADase and ARTase, but had little effect on the *Km* for actin [27]. Accordingly, these observations suggest that Arg-295 plays an essential role in the binding of Ia to NAD^+^.

Substitution of Glu-378 within the E-X-E sequence with alanine in Ia resulted in a complete loss of NADase and ARTase activities [27], as reported by Perelle *et al.* [28]; however, conservative substitution of Glu-378 with aspartic acid resulted in little effect on NADase activity, and a drastic reduction in ARTase activity. These results indicate that Glu-378 plays an important role in ARTase activity, but not in NADase activity. It therefore is likely that the carboxyl group of the side chain in the amino acid at position 378 is not required for NADase activity, but is essential for ARTase activity. Barth *et al.* [29] and Radke *et al.* [31] found that Glu-378 within the E-X-E motif (Glu378-X-Glu379) of C2 toxin and Glu-379 within the motif Glu379-X-Glu381 of ExoS were essential for ARTase activity, but not required for NADase activity. Our results support these findings. A kinetic analysis showed that replacement of Glu-378 with aspartic acid resulted in a severe reduction in the *kcat* values for ARTase activity, but had little effect on the *Km* values for NAD^+^ and actin, suggesting that the residue plays an important role in the catalytic mechanism. The glutamic acid at position 380 within the E-X-E sequence was replaced with aspartic acid and glutamine. The result shows that a conservative substitution, such as reduction of the carboxyl group at position 380 by one methylene unit or replacement of the carboxyl group by an uncharged amide, simultaneously resulted in a drastic reduction in NADase and ARTase activities. Furthermore, replacement of Glu-378 and Glu-380 with aspartic acid resulted in little effect on NADase activity and a drastic reduction in ARTase activity, respectively suggesting that Glu-380 isessential for the catalytic mechanism of ARTase. Thus, the roleof Glu-380 in Ia appears to be equivalent to that of the corresponding residues in C2 toxin and LT [27]. Damme *et al.* [32] reported the Glu-378 in Ia was photolabeled by NAD^+^, but that Glu-380 was not. Therefore, the Glu-378 and -380 residues seem to play different roles in the ADP-ribosylating activity of Ia.

Cieplak *et al.* [33] reported that substitution of Glu-112 (E110-X-E112) in LT resulted in a marked reduction in ARTase activity, suggesting that the residue plays a specific role in the mechanism of ADP-ribosylation and represents an essential catalytic residue. In addition, they suggested that Glu-110 is unlikely to play a specific role in the reaction mechanism. Hara *et al.* [34] reported that rat T-cell antigen RT 6.1 (Q207-X-E209) catalyzes NADase, but not transfer of the ADP-ribosyl moiety and that a mutant RT 6.1 in which Gln-207 was replaced with glutamic acid exhibited ARTase activity. Our result is consistent with their findings in that the first glutamic acid residue in the E-X-E motif is essential for ARTase activity, but not for NADase activity [27]. However, C3 and EDIN in the ADP ribosyltransferase family, which ADP-ribosylates small GTP binding proteins of the rho family, have a glutamine residue in the motif, suggesting that the residue in C3 and EDIN which correspond to the residue at position 380 in Ia is not required for ARTase activity [35]. Thus, the residue in the motif may depend on the substrate.

The replacement of Ser-338 with alanine or cysteine was not entirely effective, suggesting that the hydroxyl group of Ser-338 is not essential for these activities [27]. Furthermore, the substitution of Ser-338 with amino acids having a large side chain completely disturbed the ARTase activity. Thus, Ser-338 may be extremely close to the catalytic site. Barth *et al.* [29] reported that Ser-348 in the motif S348-T-S350 in C2 toxin plays an essential role in NAD^+^- binding or catalysis. There is no discrepancy between our results and their model. Replacement of Thr-339 or Ser-340 with alanine resulted in a significant but not severe reduction in NADase and ARTase activities. Replacement of Thr-339 and Ser-340 did not result in a marked reduction in the *Km* and *kcat* values for NADase or ARTase, compared with that of Ser-338. Therefore, it is possible that these residues do not play an important role in binding and catalytic reactions [27].

**Figure 5:**
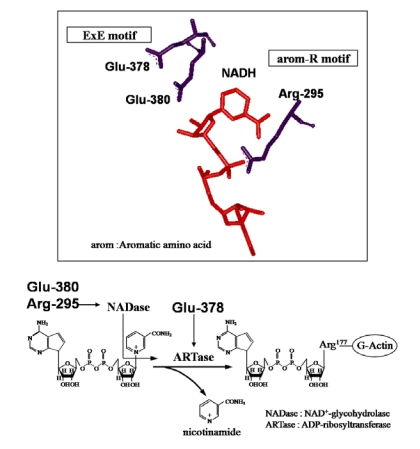
Amino acid residues at the active site of Ia.

These observations indicate that Ia has both NADase and ARTase activities; the former transfers the ADP ribose moiety to a water molecule, and the latter transfers the moiety to actin [5]. Thus, it seems that Arg-298 and Glu-380, required for these activities in Ia, play an important role in the cleavage of the *N*-glycosidic bond of NAD^+^ and that Glu-378, required for ARTase activity, but not for NADase activity, is essential for the transfer of the ADP-ribose moiety to actin, confirming that Ia belongs to the CT group in the ADP-ribosylating enzyme family, not the DT group [27] (Figure 5).

**Figure 6:**
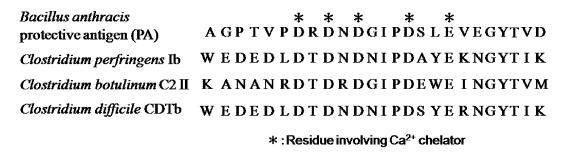
Amino acid sequence in the region containing the calcium-binding motif in members of the binary toxin family.

The crystal structure of PA [36] and C2II [37] similarly reveals four domains in these components. Gupta *et al.* [38] suggested that Ca^2+^plays a role in maintaining the conformation of PA, based on an analysis of the relationship between Ca^2+^ and PA. Gao-Sheridan *et al.* [39] reported that the bound Ca^2+^ in PA plays a role in maintaining the conformation of an *N*-terminal region designated domain I which allows PA63 to oligomerize and bind to the enzyme components. It also is speculated that the *N*-terminal region of the binding component has five conserved Asp residues and one conserved Glu residue whose side-chain carboxyl groups chelate two calcium ions (Ca^2+^-binding motif) [40] (Figure 6). It was reported that the N and C termini in the binding components represent the docking site for the enzyme component and the binding site for the cell, respectively [5]. We reported that Ib binds to a receptor on MDCK cells, forms a heptamer, and then the oligomer is internalized *via* endocytosis [41]. The replacement with alanine of Asp-8, -10 and -12, which are included within the conserved motif in Ib, led to a severe reduction in cytotoxic activity [42]. The internalization of these variants in cells without Ia resembled that of wild-type Ib alone [42]. We also reported that Ia bound to Ib in lipid rafts is internalized by endocytosis [43]. However, Ia is not internalized in the cells in the presence of D8A, D10A and D12A, suggesting that the Ca^2+^-binding motif of Ib has an important role in the interaction of Ia in the presence of Ca^2+^ [42]. Gao-Sheridin *et al.* [39] reported that Ca^2+^ within domain I of PA is required for PA63 subunit-subunit interactions. Gupta *et al.* [38] speculated on the basis of the replacement of amino acid residues in the Ca^2+^-binding motif of domain I of PA, that the motif is critical for initial protein assembly. However, substitutions at Asp-8, -10 and -12 in Ib resulted in about half as much oligomer in cell membranes, indicating that the residues in the Ca^2+^- binding motif of Ib did not greatly affect the oligomerization of Ib [42].

**Figure 7:**
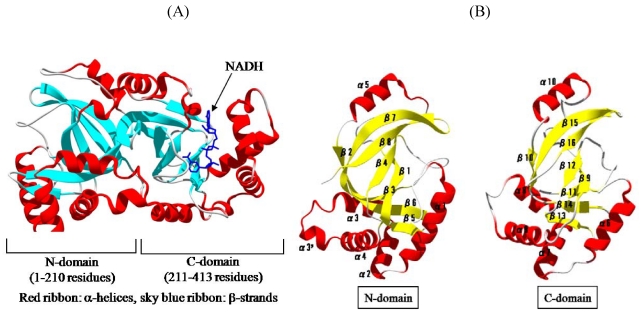
Structure of Ia. (A) Structure of Ia with NADH (B) The *N*-domain and *C*-domain of Ia.

We solved the crystal structure of Ia with NADH, which acts as a inhibitor of the enzymatic activity of Ia, at a resolution of 1.8 Å [44,45]. The 411 amino acid residues of Ia were folded into a mixed α/β structure of dimensions 68 Å × 50 Å × 47 Å. The enzyme is divided into two domains, the *N*-domain (residues 3-210) and the *C*-domain (residues 211-413) (Figure 7A). These domains are structurally similar despite limited sequence identity. Each domain consists of a β-sandwich core formed by a five-stranded mixed β-sheet (β1, β4, β8, β7, and β2 in the *N*-domain; β9, β12, β16, β15, and β10 in the *C*-domain) and a three stranded anti-parallel β-sheet (β3, β6, and β5 in the *N*-domain and β11, β14, and β13 in the *C*-domain) (Figure 7B). Around the β-sandwich core, consecutive α-helices (α1, α2, α3, α30, and α4 in the *N*-domain and α6, α7, α8, and α9 in the *C*-domain) and one additional α-helix (α5 in the *N*-domain and α10 in the *C*-domain) are arranged. The *C*-domain has a large cleft, which NAD binds, similar to that of the *N*-domain (Figure 7B). The structure of VIP2 and C2I was solved [46,47]. VIP2 and C2I were divided into two domains like Ia. The count of identical residues in these enzyme components was 20% in the *C*-terminal domain and only 10% in the well conserved *N*-terminal domain. However, when Ia was superposed on VIP2, their *C*-domains were found to be quite similar in structure, but not their *N*-domains. It has been reported that the *N*-domains of C2I and Ia interact with the binding component, C2II and Ib, respectively [5]. Therefore, it is possible that the difference in structure of the *N*-domain reflects the difference in the binding component. It appears that the *N*- and *C*-domains of these enzyme components play an important role in the binding to substrate and the binding component, and catalytic activity, respectively.

**Figure 8:**
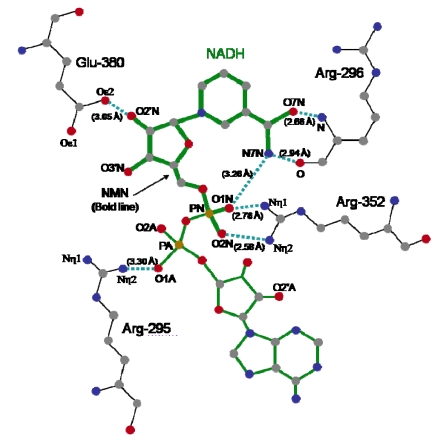
Distance between amino acid residues of the *C*-domain of Ia and NADH.

The crystal structure of Ia with NADH showed that the nicotinamide ring is clearly identifiable and nicotinamide is not cleaved [45,48]. NADH was highly folded in the cavity and formed a ring. As shown in Figure 8, N7N of the carboxyamide group on nicotinamide and O1N of the nicotinamide mononucleotide moiety (NMN) phosphate formed a hydrogen bond. Arg-295 formed a hydrogen bond with O1A of NADH. The Nη1 and Nη2 atoms of Arg-352 formed hydrogen bonds with O1N and O2N of NADH, respectively. Oε2 of Glu-380 formed a hydrogen bond with O2’N of the nicotinamide ribose in the NMN. All of these hydrogen bonds appear important in making the NMN conformation compact and ring-like [45,48].

Residues located near the NADH-binding site in the *C*-domain of Ia have structural similarity to those in the *C*-domain of VIP. Tyr-246, Tyr-251, Asn-255, Arg-295, Glu-301, Ser-338, Phe-349, Arg-352, Glu-378, and Glu-380 in Ia are conserved, compared with those in VIP. A kinetic analysis of amino acid residues in the cavity showed that Tyr-251, Phe-349 and Arg-352 in Ia are required for binding to NAD^+^[45,48]. Thus, they are considered essential for ADP-ribosylating activity. One difference in the catalytic *C*-domain between Ia and VIP2 is in the occluding loop (372-380) that overlaps the so-called ARTT motif (373-PGYAGEYE-380), which includes the EXE motif (Glu-378 and Glu-380). Glu-378 and Glu-380 on the loop play an essential role in the catalytic activity of ADP-ribosylation [27], as mentioned above. While Glu-380 in Ia had the same conformation as that in VIP2, the orientation of Glu-378 located on the flexible loop differed between Ia and VIP2. Furthermore, NAD^+^-induced conformational change of the ARTT motif was seen in C3 toxin, which ADP-ribosylates Rho [49,50]. Therefore, it is likely that the ARTT motif is important for recognition of substrates.

When the *C*-domain of Ia was superimposed on that of DT, the β-sheet fitted that of DT well, but the α-helical part fitted less well, and amino acid residues in the catalytic site of Ia were superposed on those of DT [48]. Therefore, it appeared that Arg-295, Glu-380 and Phe-349 in Ia are arranged almost identically to His-21, Glu-148 and Tyr-65 in DT, and the STS motif near the folded NAD^+^ in Ia is replaced by a YST sequence in DT [45,48]. From these findings it is concluded that toxins with ADP-ribosylating activity have a common basic tertiary structure consisting of a β-sandwich.

**Figure 9:**
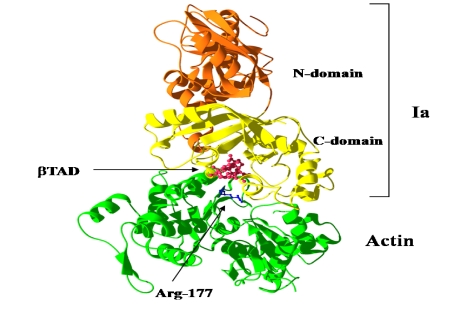
Ia-actin-TAD complex.

**Figure 10:**
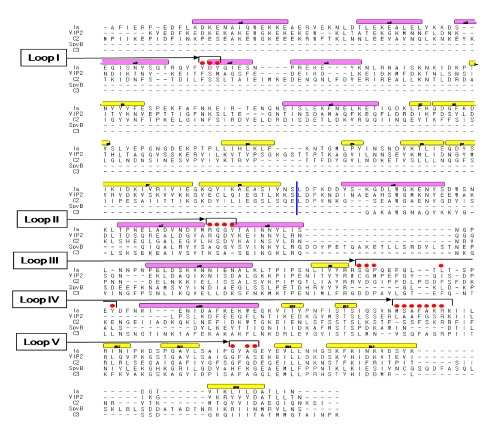
Sequence alignment of ADP-ribosylating toxins. Red circle: Amino acid residues in the expected actin binding region. Blue line: interface between the N and C domains. Secondary structure elements are coded as follows, β sheet: yellow bars, α helices: magenta bars.

**Figure 11:**
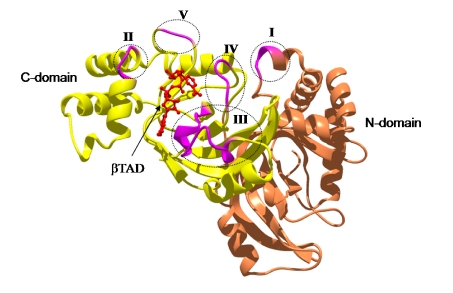
The loops in Ia involved in recognizing actin. Roman numerals (I-V) show the five actin-binding loops in Ia.

We reported the crystal structure of Ia-actin in a complex with the nonhydrolyzable NAD analog βTAD, at a resolution of 2.8 Å [51] (Figure 9). Arg-177 and Asp-179 in G-actin are in close proximity to βTAD and Glu-378 of Ia. The shape of bound βTAD is similar to that of NADH, which assumes an NMN ring-like conformation. The structure indicated that Ia recognized actin *via* five loops around NAD^+^: loop I (Tyr-60-Tyr-62), loop II (active-site loop), loop III, loop IV (PN loop), and loop V (ADP-ribosylating turn-turn loop) (Figure 10 and Figure 11). Furthermore, Glu-378 in the EXE is in close proximity to Arg-177 in actin. 

**Figure 12:**
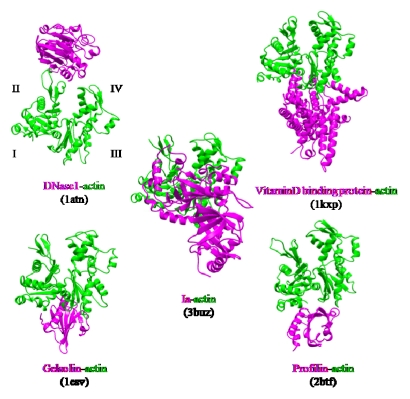
Comparison of the molecular recognition interface of Ia and actin-binding proteins. Actin (green) and Ia (magenta) were shown.

The actin-binding interface of Ia was compared with that of other actin-binding proteins, including gelsolin [52], vitamin D-binding protein [53], DNaseI [54], and profilin [55]. DNaseI binds with high affinity to subdomains II and IV of actin, whereas the other three proteins bind to subdomains I and III. Ia mainly binds to actin through subdomains I, III, and IV, which cover its surface in the region that includes Arg-177 (Figure 12). The structure of the actin-Ia-βTAD complex showed that the recognition interface of Ia differes from that of other known actin-binding proteins [51].

We investigated the role of amino acid residues, Asp-61, Tyr-62, Arg-248, Tyr-306 and Tyr-311, at the interface between Ia and actin [51]. The replacement of Tyr-62 in loop I and Arg-248 in loop II with alanine resulted in a drastic reduction of ARTase activity, but not NADase activity. However, the replacement of Asp-61 in loop I and Tyr-306 and Tyr-311 in loop III with alanine had little effect on ADP-ribosylating activity and cytotoxicity. Deletion of the N domain caused a drastic decrease in ARTase activity, whereas >80% of the NADase activity was retained, suggesting the *N* domain of Ia to be important for binding to actin. From these results, it was concluded that loops I and II of Ia play an important in binding to actin. On the other hand, the structure of actin within the complex was relatively unchanged from its monomeric form. Furthermore, binding of Ia to actin induced a subtle shift of loops II, IV, and V of Ia, suggesting that these loops play an important role in the recognition of actin. Therefore, it appears that actin is recognized by loops III, IV and V of Ia through not only ionic interaction, but also van der Waals interaction, based on the complementary shape. This feature might be common to all type IV ADP-ribosylating toxins that consist of two domains [51].

**Figure 13:**
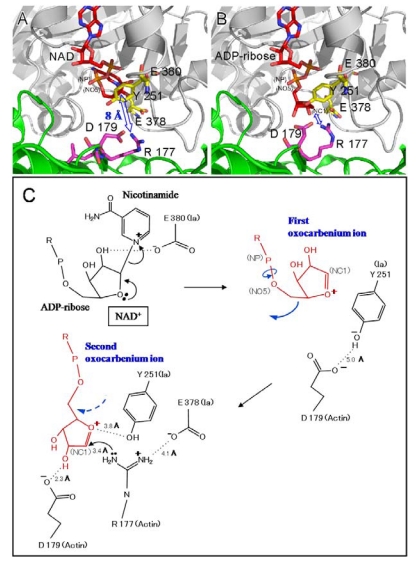
Mechanism for ADP-ribosylation of actin by Ia.

Two different mechanisms could be considered for the ADP-ribosylation of Arg-177 in actin by ADP-ribosylating toxins, the SN1- and SN2-reactions [45,46]. The positively charged Arg-295 and Arg-352 interact electrostatically with a phosphodiester group of NAD^+^ and contribute to the highly folded conformation of NMN. The specific conformation appears to induce a shift in equilibrium toward the production of oxocarbenium cations from NAD^+^. A crystallographic analysis of the Ia-actin-βTAD complex showed the distance between NC1 of *N-*ribose and the guanidyl nitrogen of Arg-177 to be > 8 Å (Figure 13A), which might make a direct nucleophilic attack impossible *via* the SN2 reaction. Even if the reaction proceeds *via* the SN1 mechanism, it is unclear how this distance is shortened. Therefore, it appears reasonable that the SN1 reaction occurs *via* the production of two intermediates: an oxocarbenium ion intermediate and a cationic intermediate (Figure 13B) [51]. After the oxocarbenium ion intermediate is produced, the NP-NO5 rotation of ADP ribose permits the *N-*ribose access to Arg-177. This second cationic intermediate allows the NC1 of *N-*ribose to approach the guanidyl nitrogen of Arg-177 to within a distance of 3.4 Å. (Figure 13B and C). In this model, Asp-179 of actin plays a stabilizing role by making contact with the *N-*ribose 2’ OH (Figure 12B and Figure 12 C). Finally, the nucleophile Arg-177 of actin, which associates with Glu-378 of Ia, attacks NC1 of the oxocarbenium ion, leading to ADP-ribosylation. We reported that Tyr-251 binds to actin [45]. The structure shows that this residue is in close proximity to Asp-179 of actin. It therefore appears that Tyr-251 guides the NP-NO5 rotation to position the *N-*ribose. Furthermore, Tyr-251 might play a role in catching the oxocarbenium ion before it is transferred to the guanidyl nitrogen of Arg-177. Interestingly, it is possible that the residues around the *N-*ribose create a negative environment to protect the oxocarbenium cation and to prevent an unfavorable reaction with water; these comprise Tyr-246, Tyr-251, Ser-338, Tyr-375, Glu-378, and Glu-380. In addition, there is an open space within the interface of the complex, suggesting that it does not prevent reactions with water. The space makes it possible to rotate *N-*ribose, thereby allowing NC1 of the oxocarbenium ion to come sufficiently close to react with the guanidyl nitrogen of Arg-177.

We propose a SN1 mechanism for the Arg-177 ADP-ribosylation that involves the following three steps: generation of the first oxocarbenium intermediate by the cleavage of nicotinamide, rotation to form the second cationic intermediate around the NP-NO5 bond through the release of the conformational strain, and nucleophilic attack of Arg-177 activated by the interaction with Glu-378 [51].

## 4. Mode of Action

Administration of Ib to mice given anti-Ia antiserum within 120 min after the intravenous injection of Ia did not cause death [11]. The result shows that the anti-Ia antiserum reacted with Ia *in vivo* until at least 120 min after the intravenous injection of Ia, suggesting that Ia remains free in the blood stream in the absence of Ib. Administration of Ia to mice given anti-Ib antiserum 5 min after the injection of Ib resulted in death, indicating that the antiserum was unable to neutralize the activity under the conditions. It therefore appears that once it binds to the receptor, Ib proceeds immediately to a stage which can no longer be inhibited by the antiserum. These results suggest that Ia binds to the oligomer of Ib and penetrates the target tissue [11]. Dermonecrosis was observed at the site of injection of Ib, but not Ia, when Ia and Ib were injected intradermally at separate sites in guinea pigs. Furthermore, the intraperitoneal injection of Ia and Ib after the intradermal injection of Ib and Ia, respectively, resulted in dermonecrosis at the site of intradermal injection of Ib, but not the intradermal injection of Ia. These results indicate that Ia is free in the blood-stream and Ib binds to a receptor in the skin, suggesting the migration of Ia to the site of injection of Ib to be essential to the dermonecrotic activity induced by separate injections of the two components [11]. Accordingly, it appears that Ia binds to Ib bound to target tissues, resulting in lethal effects and dermonecrosis. 

The receptor-bound PA is reported to be internalized by receptor-mediated endocytosis [56]. Milne *et al.* [57] found that PA formed oligomers during the intoxication of mammalian cells. The Aktories group has reported that C2II toxin, which resembles PA, forms an oligomer during intoxication [58]. However, it has been reported that Ib possesses no activity [6,9,59]. Knapp *et al.* reported that Ib formed a single-channel in artificial lipid bilayers and Vero cells [60]. Recently, we reported that Ib specifically binds to Vero cells and induces the release of K^+^ from the cells in a dose- and time-dependent manner, showing that Ib forms ion-permeable channels in Vero cells without causing death [41]. Therefore, it is apparent that Ib also possesses biological activity. SDS-PAGE revealed that the migration of the Ib oligomer formed by incubation of Vero cells with Ib at 37 °C was identical to that of the Ib heptamer isolated from the purified Ib preparation, showing that Ib forms an oligomer on the cell membrane [41]. The time course of K^＋^’s release from the cells treated with Ib coincided with that of the oligomer’s formation. On the other hand, when Vero cells were incubated with oligomers isolated from the purified Ib preparation, no release of K^+^ was detected, rounding of the cells was not observed in the presence of Ia, and the oligomers bound to the cells were hydrolyzed by treatment with pronase. The oligomer formed from the monomer in membranes of the cells was not hydrolyzed by the enzyme. These observations imply that the monomer of Ib binds to the cell and forms a functional oligomer in the membrane [41].

PA and diphtheria toxin were shown to be translocated into the cytosol from an endosomal compartment. C2 toxin was reported to enter cells through receptor-mediated endocytosis [59]. Furthermore, Barth *et al.* [58] reported that C2 toxin is released into the cytosol after acidification of the endosomal compartment. Considine and Simpson [59] suggested the internalization of Ib by receptor-mediated endocytosis to be essential to the toxicity of iota-toxin. Blocker *et al.* reported that treatment with Ib plus Ia in the presence of bafilomycin, methylamine and ethylamine, which prevents acidification of endosomal vesicles, caused no change in cells and, in addition, did not cause ADP-ribosylation of actin in the cytosol [61]. Ib was trans-cytosed and permanently exposed on surface of the opposite cell or continuously recycled between an endosomal compartment and the cell surface [23]. We obtained evidence of the cellular routing of Ib. After Vero cells were treated with these inhibitors, Ib induced the release of K^＋^from the cells, and the oligomer formed on the cells and was inserted into the endosomal membranes. These observations suggest that Ib forms ion-permeable channels in the membranes (functional oligomer) and that Ib is endocytosed after binding to the cell in the absence of Ia.

Recently, lipid rafts have been reported to act as surface platforms during signal transduction and endocytosis [62]. In addition, some toxins [63,64], bacteria [65], and viruses [66] have been shown to enter cells *via* lipid rafts. We investigated whether or not the oligomer of Ib accumulates in lipid rafts and Ia bound to the oligomer enters the cell. The monomer of Ib was detected in the Triton X-100-soluble and -insoluble fractions, the whole membrane, of cells incubated with Ib at 4 °C, suggesting that the receptor of Ib is distributed around cytoplasmic membranes. Thus, it is unlikely that the receptor is confined to lipid rafts. The oligomer of Ib was detected in lipid rafts after incubation of the washed cells at 37 °C, suggesting that Ib bound to the receptor is gathered in lipid rafts, and the oligomer is formed there. In addition, the *C*-terminal region of Ib (Ib421-664) blocked the binding of Ib to the cells, as reported by Marvaud *et al.* [67], and was detected in lipid rafts from MDCK cells. It therefore appears that the *C*-terminal region of Ib binds to a receptor that is distributed over the entire membrane of the cell, that a receptor which is linked with Ib gathers in lipid rafts at 37 °C, and that Ib forms oligomers in the lipid rafts [43]. The treatment of MDCK cells with MβCD reduced the cholesterol content of lipid raft fractions, the binding of Ib to the cells, and the rounding activity induced by Ia plus Ib. Therefore, it has been speculated that the functional properties of lipid rafts that are relevant to the intracellular trafficking of Ib may be especially susceptible to treatment with MβCD. It was reported that the disruption or depletion of membrane-associated cholesterol causes major changes in the function and/or distribution of raft-associated membrane components [68]. It therefore appears that a function of lipid rafts is to gather Ib so that it forms oligomers and enters into cells [43].

The internalization of iota-toxin is mediated through lipid rafts (cholesterol-rich microdomains) at the plasma membrane, suggesting that the lipid rafts contain all of the components necessary for the mediation of the toxin’s endocytosis. Gibert *et al.* reported that after internalization, it was found that iota-toxin was not routed to the Golgi apparatus and translocation of the Ia occurs early endosomes [69]. The findings that Ib is concentrated in lipid rafts, where it forms oligomers, and that it induces endocytosis have provided useful information on the cytotoxicity induced by bacterial toxins.

## 5. Conclusions

**Figure 14:**
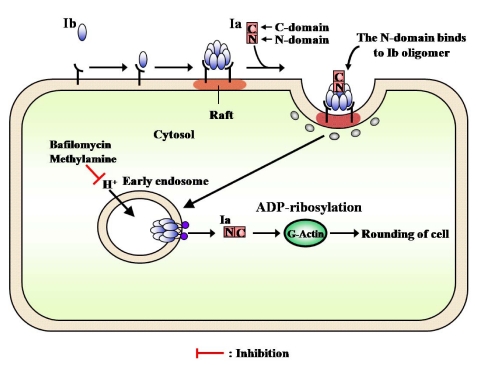
Model for internalization of iota-toxin into cells.

Iota-toxin enters host cells and induces toxicity by exploiting the cell’s endogenous pathways as follows (Figure 14). (1) Ib binds to a receptor at plasma membranes, then moves to lipid-rafts and forms a heptamer, (2) the *N*-terminal domain of Ia binds to the Ca^2+^-binding motif in the *N*-terminal region of Ib, (3) Ia bound to the Ib oligomer is internalized in cells by receptor-mediated endocytosis, (4) the complex of Ia and Ib is transported to the early endosomes, where acidification promotes cytosolic entry of Ia, and 5) the *C*-domain and part of the *N*-domain of Ia bind to G-actin in the cytosol and ADP-ribosylate it, thereby blocking the polymerization of actin, and eventually intoxicating cells.
